# The origins of molecular psychiatry

**DOI:** 10.1186/2049-9256-1-3

**Published:** 2013-04-23

**Authors:** Eric J Nestler

**Affiliations:** The Mount Sinai Department of Neuroscience, Mount Sinai School of Medicine, One Gustave L. Levy Place, New York, NY 10029-6574 USA

One day, in 1987, Ron Duman and I were setting up our new laboratories in CMHC, the Connecticut Mental Health Center, a state mental hospital jointly run by the State of Connecticut and Yale University. Since the 1960’s, CMHC was unique as a state mental hospital in that it included a basic neuroscience research group embedded within inpatient and outpatient units that care for the area’s mentally ill. Duman and I were newly hired assistant professors and our appointments at Yale were made possible by the conversion of a basement corridor, which had heretofore been used to teach psychiatry residents group and family therapy, into biochemical laboratories. As Duman and I were setting up our new labs, we decided to name our corridor “The Laboratory of Molecular Psychiatry,” and we had the gumption to post a huge sign with this new name, which we had made at a local photocopying store, without any permission from our higher-ups. Thus was born the field of Molecular Psychiatry!

The name Molecular Psychiatry was particularly provocative in 1987. One of our fellow faculty researchers, a behavioral neuroscientist, posted a sign on his laboratory, “Laboratory of Molecular Fear.” Another faculty member in the department labeled his office, “Laboratory of Molecular Psychoanalysis.” I’m not sure what was most discomfiting about Molecular Psychiatry. The name made eminent sense to Duman and me, and our primary mentors at Yale, George Heninger and George Aghajanian. The 1980’s marked the dawn of the molecular revolution in medicine and we all believed that brain diseases that manifest themselves in behavioral abnormalities would prove to be no different from diseases of all other organ systems, namely, that psychiatric disorders have fundamental molecular lesions that need to be identified and understood and, with that knowledge, reversed or prevented.

Duman and I, and our many colleagues at Yale and across the country and world, set out to establish the molecular foundations of psychiatry. This molecular approach to psychiatry depended on the tremendous advances in biological psychiatry and neuropharmacology that had taken place the previous several decades. These advances included the development of animal models of particular mental disorders as well as an increasingly sophisticated understanding of the brain’s neurotransmitter systems and their regulation by all major classes of psychotropic drugs. The goals of Molecular Psychiatry, as we first formulated them, are several fold. First, the field aims to identify abnormalities in particular neuronal cell types which account for the behavioral pathology seen in the animal models of psychiatric disorders A second goal is to understand, at the molecular and cellular levels, how psychotropic drugs produce their clinically relevant actions and reverse the behavioral pathology in the animal models. A third goal is to understand how the genetic underpinnings of an individual animal combine with a host of environmental factors to determine the individual’s inherent vulnerability for developing that behavioral pathology. The fourth and ultimate goal is to translate this information to clinical populations: to identify the genetic variants that help determine individual risk for mental illness, to study the molecular pathophysiology of mental disorders in humans, and to use the increasing knowledge of this pathophysiology to develop improved treatments, even preventive measures, for these conditions.

Although first greeted with disbelief and even some snickers, we achieved several milestones in those early years that helped establish Molecular Psychiatry as a bona fide field. Duman and I organized several symposia for the American College of Neuropsychopharmacology and the American Psychiatric Association in the early 1990’s. In 1993, I published a book, *The Molecular Foundations of Psychiatry*, with Steve Hyman, then at Harvard and one of our fellow pioneering molecular psychiatrists. Hyman and I also organized a lead symposium at the Society for Neuroscience meeting in 1995 on Molecular Psychiatry which was widely attended. Duman, Hyman, and I, alone and in various combinations, along with many colleagues in the field, also published several articles in the mid-1990’s that defined this burgeoning new field and laid out its promise for the future. By the late 1990’s the field had matured to a point where a new journal, *Molecular Psychiatry*, was formed by the *Nature* publishing group. Molecular Psychiatry made it!

Despite this promise for the field, the past two decades have been very frustrating. We have learned a great deal about the molecular actions of psychotropic drugs and stress in animal models, but we have not yet been able to use this information to develop new diagnostic tests (biomarkers) or improved treatments for mental illness. Furthermore, only in the past 2–3 years have we begun to identify *bona fide* genetic variations that confer risk for psychiatric disease in people, and currently we have discovered well less than 10% of the genetic causes of these complex syndromes. Nevertheless, we remain optimistic that molecular approaches will lead to revolutionary advances in psychiatry—the brain and its disorders are simply far more complicated than we ever imagined. An impressive array of new and extremely powerful experimental approaches, in genetics, epigenetics, optogenetics, multi-unit recording from awake, behaving animals, and brain imaging, among others, should set the pace for accelerating advances over the next decade. We hope and expect that we will at long last witness the molecular revolution fully transforming the field of psychiatry into a modern medical subspecialty.

In this context, I greatly welcome the foundation of the Journal of Molecular Psychiatry (JMP), because its modern open-access format will further disseminate and strengthen of the idea of molecular psychiatry.

